# Latin American Autoimmunity and the Brazilian Anthropophagy

**DOI:** 10.1080/17402520600876820

**Published:** 2006

**Authors:** R. A. Levy, V. Pordeus

**Affiliations:** 1 Rheumatology Discipline Universidade Estadual do Rio de Janeiro Rio de Janeiro RJ Brazil; 2 Rio de Janeiro & Rheumatology Discipline Hospital Pró-Cardíaco Universidade de São Paulo São Paulo SP Brazil


*Clinical & Developmental Immunology*, June–December 2006; 13(2–4): 79–80

Taylor & Francis

Taylor & Francis Group

**EDITORIAL**

### Latin American autoimmunity and the Brazilian anthropophagy

R. A. LEVY[1] & V. PORDEUS[2]

[1]*Rheumatology Discipline, Universidade Estadual do Rio de Janeiro, Rio de Janeiro, RJ, Brazil, and* [2]*Rio de Janeiro & Rheumatology Discipline, Hospital Pró-Cardíaco, Universidade de São Paulo, São Paulo, SP, Brazil*

> "How to explain, in an underdeveloped country, the appearance of an avant-garde and justify it not as symptomatic alienation, but as a decisive factor in collective progress?"
>
> Helio Oiticica, 1967.

At the end of the 1960s a movement in Brazilian culture bloomed, it manifested itself in several sectors, such as visual arts, poetry, cinema and theater, with consequences and repercussions in the whole national panorama and the future of the own country's thinking. It was an avant-garde, culturally exuberant, original and inspiring movement, seen now as a cultural revolution: the *tropicalism*. The name was given after the installation "Tropicália" exhibited by the artist Helio Oiticica in the Rio de Janeiro Museum of Modern Art in 1967 (Basualdo 2005). All happened in spite of the violent military dictatorship which has tortured, killed, exiled or repressed most of the local *intelligentsia*. Among the many references of tropicalism, one stood for its great importance: "The Anthropophagic Manifesto" written by Oswald de Andrade in 1928, as an echo of the seminal modernist week of art that happened in 1922 in São Paulo. De Andrade proclaimed anthropophagy as the basic principle for the construction of Brazilian identity, to devour myths and ideas that prevent this country from confronting its reality and inventing its history, to rethink its own cultural identity (de Andrade 1928). He evoked the history of the Caraíba tribe of Brazilian Indians who had the practice of eating enemies killed in their territory (Carneiro da Cunha 1992).

The second Latin American Congress of Autoimmunity reminded us on the Latin-American scientific originality, quality, and also here, the existence of an improbable avant-garde in an underdeveloped country, better, in an underdeveloped continent; allowed us witness the "Anthropophagy" in science and medicine. For instance, shown in the heterodox thinking of the Brazilian immunologist Nelson Vaz, a tropicalistic scientist, who back in the 70s built up his identity through the anthropophagy of mainstream immunology and elaborated an original theory, *The Conservative Physiology of the Immune System* as can be seen in this issue.

The innovative work of J-M Anaya and his group from Medellin, Colombia, investigating genes and polymorphisms, propose a new look into autoimmune diseases, finding common pathways, to clinically distinct entities.

Rescuing rheumatic fever from neglect, L Guilherme and collaborators from São Paulo, Brazil showed us their struggle for an effective vaccine against this disease that still affects around 30 million of people around the (mainly underdeveloped) world.

Also originally, Ana Faria from Belo Horizonte, Brazil had presented elucidating mechanisms on the still promising oral and nasal tolerance therapy for autoimmune as well as allergic diseases; she emphasized the "indirect effects" of oral tolerance as an alternative way to applying this therapy, as can be seen in her manuscript.

Luís Javier-Jara from Mexico City, discussed the neuro-endocrine interactions in immune system, presenting his instigative data with autoimmune disease patients, reminding us on those fundamental aspects of autoimmunity as can be read in his work.

Melanie Rodacki from Rio de Janeiro, Brazil showed new aspects on the autoimmune process of Type1 diabetes, revealing abnormal patterns of natural killer cells in collaboration with the Joslin

Correspondence: R. A. Levy, Rheumatology Discipline, Universidade Estadual do Rio de Janeiro, Rio de Janeiro, RJ, Brazil.

ISSN 1740-2522 print/ISSN 1740-2530 online © 2006 Taylor & Francis
DOI: 10.1080/17402520600876820

Diabetes Center from Harvard University. Shedding light into the role of these cells in the autoimmune pathophysiology as discussed in her paper.

We are very happy that during the congress we could see the exceptional work of Latin-American scientists: more than this, we had with us the brightness and friendship not only from our own continent, but also from other continents. The list is headed by Yehuda Shoenfeld and Ricard Cervera, great encouragers and bright minds that know how to translate science into daily practice and other likewise special friends along with them, not less supportive and brilliant: M Eric Gershwin, Stanly Naguwa, Edward Chan from USA, Ronald Asherson from South Africa, Anat Achiron from Israel, Jordi Lopez from Spain, Ivan Foeldvari, from Germany, Georg Wick from Austria, all added in consistency and original information on several areas. We could see that myths are delusions and the questions pour faster than the answers.

Fundamentally, the construction of the Latin-American science, the anthropophagy of ideas and data rethinking our identity and ways of experimenting, is in demand.

In this special issue of *Clinical and Developmental Immunology*, so kindly dedicated to our congress by the editor M Eric Gershwin, you will find a taste of the Latin-American science and way of thinking. May the Latin American 'anthropophagy' inspire you.

### References

Anaya J-M, Gómez L, Castiblanco J. 2006. Is there a common genetic basis for autoimmune diseases? Clin Dev Immunol 13:185–195.

Basualdo Co. 2005. Tropicália: A revolution in Brazilian culture. 1 ed., São Paulo: Cosac Naify.

Carneiro da Cunha M. 1992. Images of Indians of Brasil: The XVI century. Estud Avançados 4(10):91–110.

de Andrade O. 1928. Manifesto antropofágico. Rev Antropofagia 1(1).

Faria AMC, Weiner HL. Oral tolerance: Therapeutic implications for autoimmune diseases. Clin Dev Immunol 13: 143–157.

Guilherme L, Faé KC, Higa F, Chaves L, Oshiro SE, Freschi de Barros S, Puschel C, Juliano MA, Tanaka AC, Spina G, Kalil J. Towards a vaccine against rheumatic fever. Clin Dev Immunol 13:125–132.

Jara LJ, Navarro C, Medina G, Vera-Lastra O, Blanco F. Immune-neuroendocrine interactions and autoimmune disease. Clin Dev Immunol 13:109–123.

Rodacki M, Milech A, de Oliveira JEP. NK cells and type 1 diabetes. Clin Dev Immunol 13:101–107.

Vaz NM, Ramos GC, Pordeus V, Carvalho CR. The conservative physiology of the immune system. A non-metaphoric approach to immunological activity. Clin Dev Immunol 13:133–142.

